# Molecular mechanisms of ribosomal protein gene coregulation

**DOI:** 10.1101/gad.268896.115

**Published:** 2015-09-15

**Authors:** Rohit Reja, Vinesh Vinayachandran, Sujana Ghosh, B. Franklin Pugh

**Affiliations:** Center for Eukaryotic Gene Regulation, Pennsylvania State University, University Park, Pennsylvania 16802, USA

**Keywords:** gene regulation mechanisms, ChIP-seq, chromatin, transcription factor

## Abstract

The 137 ribosomal protein genes (RPGs) of *Saccharomyces* provide a model for gene coregulation. We examined the positional and functional organization of their regulators (Rap1 [repressor activator protein 1], Fhl1, Ifh1, Sfp1, and Hmo1), the transcription machinery (TFIIB, TFIID, and RNA polymerase II), and chromatin at near-base-pair resolution using ChIP-exo, as RPGs are coordinately reprogrammed. Where Hmo1 is enriched, Fhl1, Ifh1, Sfp1, and Hmo1 cross-linked broadly to promoter DNA in an RPG-specific manner and demarcated by general minor groove widening. Importantly, Hmo1 extended 20–50 base pairs (bp) downstream from Fhl1. Upon RPG repression, Fhl1 remained in place. Hmo1 dissociated, which was coupled to an upstream shift of the +1 nucleosome, as reflected by the Hmo1 extension and core promoter region. Fhl1 and Hmo1 may create two regulatable and positionally distinct barriers, against which chromatin remodelers position the +1 nucleosome into either an activating or a repressive state. Consistent with in vitro studies, we found that specific TFIID subunits, in addition to cross-linking at the core promoter, made precise cross-links at Rap1 sites, which we interpret to reflect native Rap1–TFIID interactions. Our findings suggest how sequence-specific DNA binding regulates nucleosome positioning and transcription complex assembly >300 bp away and how coregulation coevolved with coding sequences.

## Abstract (precis)

The 137 ribosomal protein genes (RPG) of *Saccharomyces* provide a model for gene coregulation. Reja et al. examine the positional and functional organization of their regulators (Rap1, Fhl1, Ifh1, Sfp1, and Hmo1), the transcription machinery (TFIIB, TFIID, and RNA polymerase II), and chromatin at near-base-pair resolution using ChIP-exo.

*Saccharomyces* contains 78 distinct ribosomal proteins (RPs) encoded by 137 genes (RPGs): 19 single genes and 59 paralogous gene pairs. Gene pairs arose from whole-genome duplication ∼100 million years ago (Wolfe and Shields 1997). RPGs are the most highly and coordinately expressed genes in the cell (Li et al. 1999; Warner 1999) and thus are ideal for understanding mechanisms of coregulation. Their expression is coordinately activated by nutrient and growth sensing and is rapidly repressed upon stress or starvation (Gasch et al. 2000).

The tight coregulation of RPGs raises the simple expectation that the structural organization of transcription factors and the mechanism of regulation is essentially the same for all RPGs. However, this has not been demonstrated for RPGs or any other set of coregulated genes beyond the presence of a common set of regulatory factors. Differences in RPG mRNA and protein turnover, protein stoichiometry, gene copy number, and potential nonredundancy of some paralogs may create gene-specific constraints on regulation that are encoded within individual RPG promoter sequences (Zeevi et al. 2011). As such, they may be distinctly regulated. Even paralogous genes that produce identical proteins may have diverged their regulatory mechanisms via either drift or selection for reasons unrelated to coding sequence (Tanay et al. 2005; Wapinski et al. 2010). It remains unclear to what extent coregulated or even paralogous genes have similar mechanisms of regulation and transcription factor organization.

Several transcription factors are associated with RPG regulation (Jorgensen et al. 2004; Marion et al. 2004; Martin et al. 2004; Schawalder et al. 2004; Wade et al. 2004; Rudra et al. 2005; Hall et al. 2006; Zhao et al. 2006; Joo et al. 2011; Knight et al. 2014). Of these, Rap1 (repressor activator protein 1) is the clearest sequence-specific DNA-binding protein (Shore 1994), binding ∼90% of RPG promoters (Warner 1999; Lieb et al. 2001). Rap1-binding sites at RPGs predominantly occur in pairs, 5–15 base pairs (bp) apart, and with preference for one orientation (Kurtz and Shore 1991; Bosio et al. 2011; Knight et al. 2014).

Rap1 alters chromatin structure (Yu and Morse 1999) and recruits Fhl1, Ifh1, Sfp1 (hereafter FIS), and Hmo1 specifically to RPG promoters (Wade et al. 2004; Rudra et al. 2005; Hall et al. 2006; Zhao et al. 2006). Fhl1 binding also depends on Hmo1 but only where Hmo1 is normally bound (Hall et al. 2006; Kasahara et al. 2011); there may also be Hmo1-independent Fhl1 recruitment mechanisms (Zeevi et al. 2011; Knight et al. 2014), which needs to be reconciled. Fhl1 interacts with Ifh1 to activate RPG transcription, which is stimulated by the TOR and PKA signaling pathways, Sirtuins, and Sfp1 (Jorgensen et al. 2004; Marion et al. 2004; Martin et al. 2004; Schawalder et al. 2004; Wade et al. 2004; Rudra et al. 2005; Hall et al. 2006; Zhao et al. 2006; Berger et al. 2007; Xiao et al. 2011; Downey et al. 2013). The binding of Sfp1 to RPG promoters has been questionable (Jorgensen et al. 2004; Marion et al. 2004; Kasahara et al. 2007).

Stress and nutrient starvation repress RPGs by dissociating all but Rap1 and Fhl1 (Wade et al. 2004; Rudra et al. 2005). To this end, Crf1 binds Fhl1 to displace Ifh1 (Cherel and Thuriaux 1995; Mencia et al. 2002; Martin et al. 2004). Ifh1 is further inhibited when acetylated by SAGA/Gcn5 (Downey et al. 2013), and Sfp1 translocates to the cytoplasm (Jorgensen et al. 2004; Marion et al. 2004). Rap1 then acts as a repressor instead of an activator, but its mechanism remains unclear.

RPG activation by Rap1 involves recruitment of the TFIIA and TFIID components of the transcription preinitiation complex (PIC) (Mencia et al. 2002; Garbett et al. 2007; Papai et al. 2010). In vitro, Rap1 makes specific functional contacts with the TBP-associated factor 4 (Taf4), Taf5, and Taf12 subunits of TFIID (Layer et al. 2010) and perhaps Taf1 and Taf2 (Ohtsuki et al. 2010; Papai et al. 2010), but whether these contacts occur in vivo at native RPGs has not been established. Hmo1 is important for TFIID binding to RPGs (Kasahara et al. 2008). Given the 200- to 400-bp distance between Rap1-binding sites and the core promoter, the intervening DNA might be looped out, as has been visualized by electron microscopy (Papai et al. 2010). This loop may be filled with RPG-specific regulators and/or nucleosomes, with the latter reportedly regulating PIC assembly (Zeevi et al. 2011; Knight et al. 2014).

With the same set of transcription factors being associated with RPGs, it comes as a surprise that they lack a consistent repertoire of well-defined *cis*-regulatory elements beyond Rap1 sites. Nonetheless, these other elements may contribute to RPG regulation (Zeevi et al. 2011). They include poly(dA:dT) tracts, IFHL motifs (Wade et al. 2004; Bosio et al. 2011), and Fhl1 motifs (Badis et al. 2008). In addition, Sfp1 has been linked to specific sites such as AAAAWTTTT (IUPAC) (Zhu et al. 2009; Zeevi et al. 2011). A/T-rich sequences exclude nucleosomes, and so it remains unclear whether their function is in Sfp1 binding, nucleosome exclusion, or some other purpose. Hmo1, Fhl1, and Ifh1 have been implicated, more or less, in IFHL motif association, although this motif contributes little to their binding (Wade et al. 2004; Rudra et al. 2005; Hall et al. 2006; Zhao et al. 2006; Kasahara et al. 2007; Zeevi et al. 2011). The seemingly sporadic organization of motifs at RPGs along with the lower resolution of genome-wide chromatin immunoprecipitation (ChIP) assays have not yet offered a complete view of coregulation.

Recent ChIP (Kasahara et al. 2011) and ChIP-seq (ChIP combined with deep sequencing) (Knight et al. 2014) studies have defined the approximate promoter locations of RPG-specific factors but have resolution limits of ∼50–100 bp. Here we used the high-resolution ChIP-exo assay (Rhee and Pugh 2011) to map the precise organization of RPG-specific factors, PIC components, and chromatin at all RPGs. In the context of well-established signaling mechanisms that turn RPG expression on and off and the known factors that bind RPGs, we investigated the molecular mechanisms of RPG coregulation. We confirmed many prior observations associated with RPG promoter organization, except for the role of nucleosomes. A major challenge for us was the spatial deconvolution of individual subunits of complexes that have multiple points of cross-linking to DNA and can cross-link indirectly via other subunits. Our findings suggest that, in addition to the classical activator–PIC structure (Rap1/TFIID), a second Rap1 molecule uses RPG-specific factors as regulatable rulers that precisely set the +1 nucleosome position via chromatin remodelers into active and repressive positions at the transcriptional start site (TSS).

## Results

### RPG-specific factors have a well-defined spatial organization

**Figure 1.**
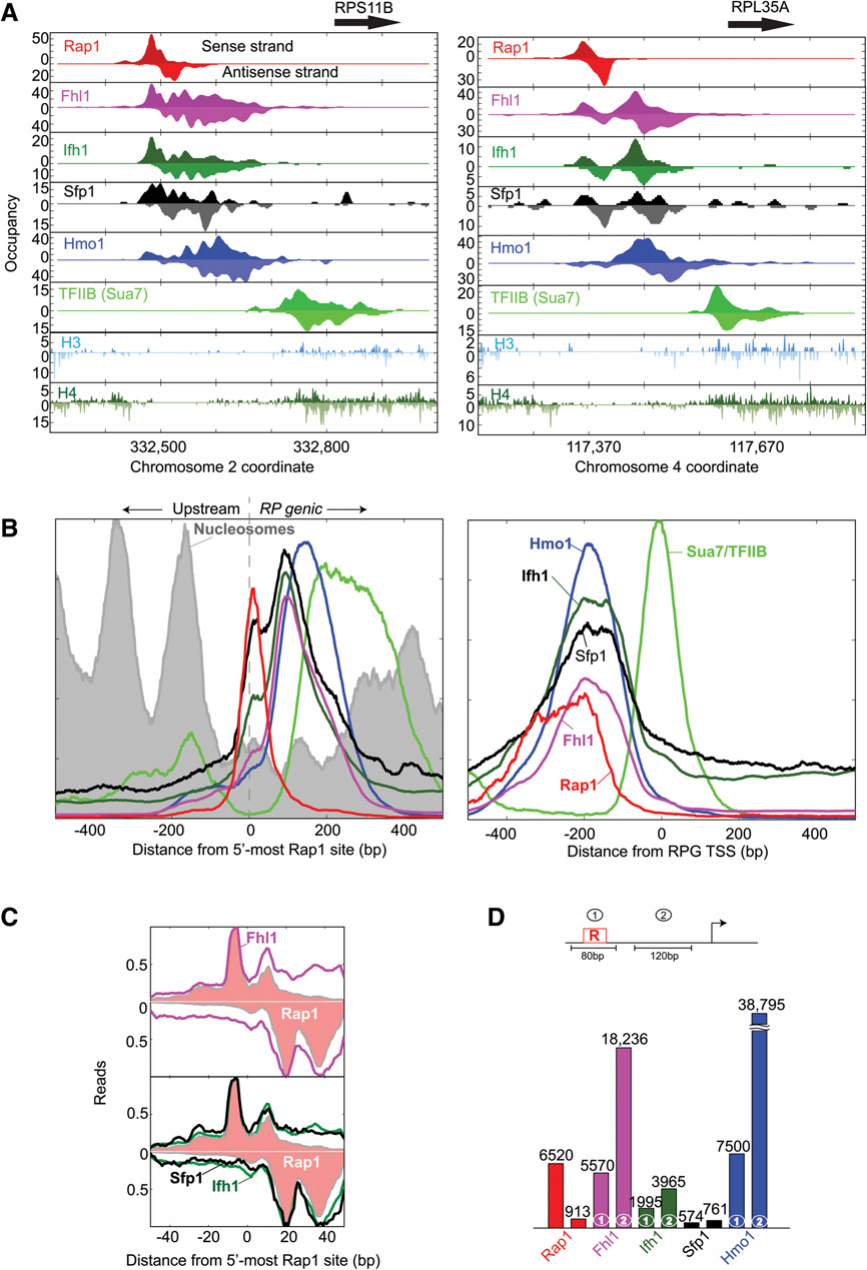
Positional organization of RPG-specific factors. (*A*) Smoothed distribution of unshifted ChIP-exo tag 5′ ends (exonuclease stop sites) on forward and reverse (inverted) strands of *RPS11B* and *RPL35A*. (*B*) RPG-averaged 5′ ends of shifted tags (representing points of cross-linking) were plotted as a smoothed frequency distribution around the most upstream Rap1-binding site (*left* panel) or their TSS (*right* panel) and oriented such that the direction of transcription was to the right. The *Y*-axis scale is linear and starts from zero but is scaled to each factor for ease of visualization. Therefore, absolute areas under the curves are not comparable. (*C*) Frequency distribution of gene-averaged (*n* = 127) unshifted tag 5′ ends for Fhl1, Ifh1, and Sfp1 (magenta, green, and black traces) compared with the equivalent for Rap1 (pink-filled plots) and oriented with the TSS to the *right*. Tags on the antisense strand are inverted. The *Y*-axis is scaled to 1 for each. (*D*) Occupancy of RPG-specific factors at Rap1 sites and the downstream regions (see the diagram). Background-normalized occupancies at Rap1 sites were calculated by summing the tag counts for each factor from −40 to +40 bp from Rap1 sites and at downstream regions from +60 to +180 bp from Rap1 sites.

The 5′ ends of ChIP-exo sequencing tags correspond to strand-specific exonuclease stop sites on cross-linked DNA molecules, as exemplified for the *RPS11B* and *RPL35A* genes (Fig. 1A). Each factor location was represented by a complex set of peaks on both strands that tended to be offset from each other by a short distance in the 3′ direction (reflecting the exonuclease “headroom”). Fhl1, Ifh1, Sfp1, and Hmo1 each displayed multiple deduced cross-linking points spread over ∼200 bp of DNA despite the potential resolution of the assay being a few base pair. The entire regulated PIC, from Rap1 to Sua7 (TFIIB) in the core transcription machinery, encompassed up to 400 bp of DNA.

The strongest positional reinforcement of RPG-specific factor patterning across all RPGs occurred when aligned by their Rap1 sites rather than their TSSs (Fig. 1B, left vs. right graphs). Upstream of Rap1, nucleosomes were positionally well organized with respect to Rap1 sites. In contrast to RPG-specific factors, the core PIC (represented by Sua7/TFIIB) was more positionally linked to the TSS than to Rap1. Thus, in addition to its known recruitment abilities, Rap1 may help position nucleosomes upstream and RPG-specific factors downstream (to the extent that they are present), whereas core promoter features may position the PIC. This is important because it had been unknown whether transcriptional regulatory complexes have fundamentally similar positional organization of subunits at coregulated genes, as this would imply similar regulatory mechanisms. Prior ChIP-seq studies on RPGs had not identified such a bifurcation of positional linkages to Rap1 and the TSS.

We therefore set out to interpret structural details of the ChIP-exo patterning, with the goal of more precisely defining the spatial and functional organization of factors at RPGs. Of the 137 RPGs, 107, 20, and 10 contain two, one, and zero Rap1-bound sites in accord with prior studies (Supplemental Fig. S1; Supplemental Tables S1, S2; Lieb et al. 2001; Zeevi et al. 2011; Knight et al. 2014). The positional organizations of Fhl1, Ifh1, and Sfp1 were nearly identical. Each displayed a major cross-linking peak centered 100 bp downstream from Rap1 and a minor peak centered over Rap1 (Fig. 1B, left panel). A zoomed-in view of the FIS patterning at Rap1 sites revealed that FIS displayed the same detailed ChIP-exo pattern as Rap1 (Fig. 1C), indicating that FIS cross-linking in this region might occur through Rap1 (i.e., “piggybacking”), although we cannot exclude that at least some cross-links are directly to the Rap1 sites. Regardless, these findings support the notion that Rap1 engages FIS.

The predominant region of FIS cross-linking was ∼100 bp downstream from Rap1 (Fig. 1B). There, Fhl1 had the highest relative levels of cross-linking (foreground/background) (Fig. 1D). This was followed by Ifh1 (4.6-fold lower) and then Sfp1 (24-fold lower). Collectively, these results suggest that Fhl1 binds more directly to promoter DNA, in accord with in vitro studies (Badis et al. 2008; Zhu et al. 2009), whereas Ifh1 and Sfp1 may bind indirectly through Fhl1 (or each other). Indirect binding was inferred (but not proven) based on their identical ChIP-exo patterning but lower signal strength. Detection of an indirectly bound protein involves more cross-links than a directly bound protein and thus has a lower yield.

### PIC and Fhl1 positioning depends on Hmo1

**Figure 2.**
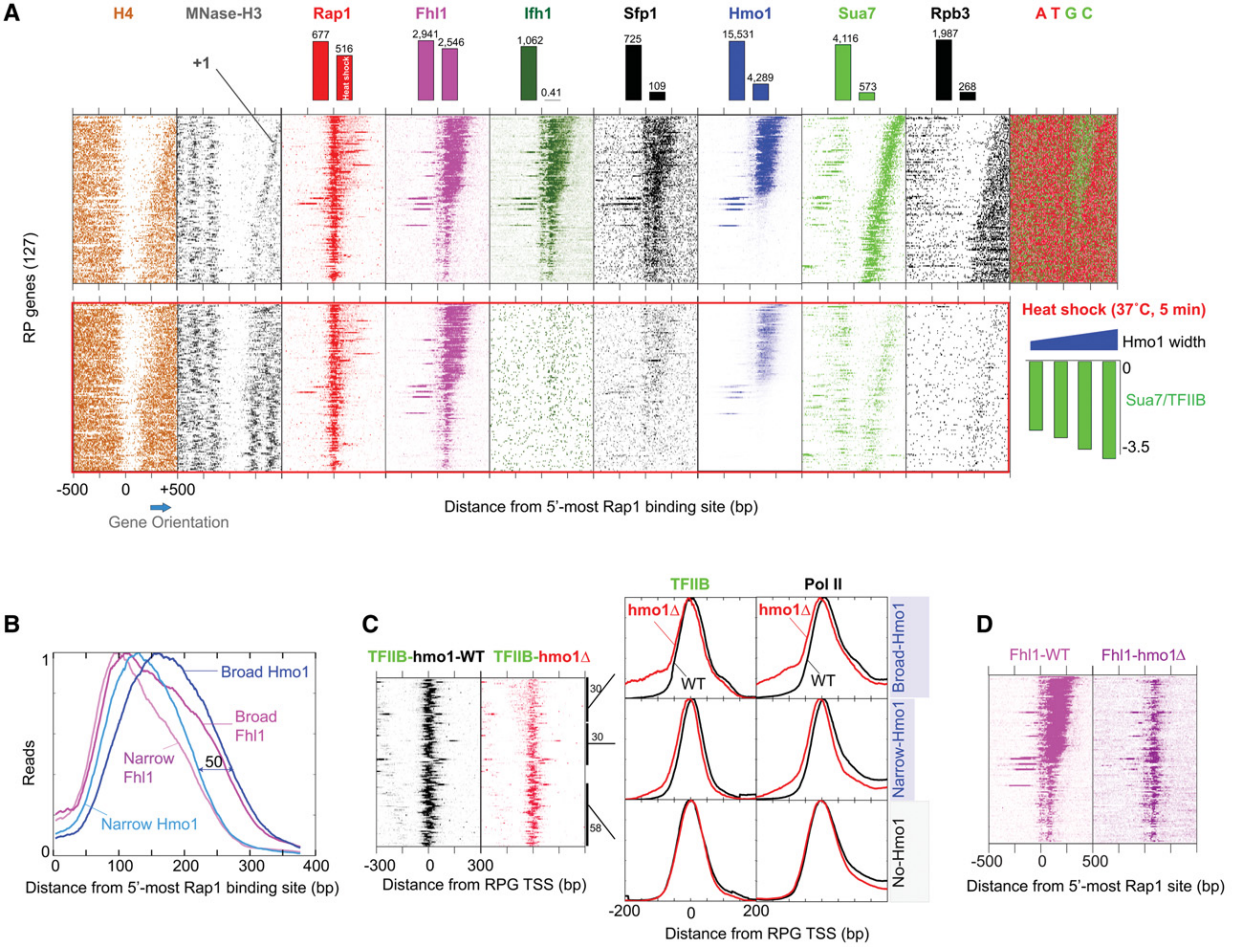
Regulation of PIC and Fhl1 positioning by Hmo1. (*A*) Heat map showing shifted ChIP-exo 5′ end tags for RPG regulators at each RPG, aligned by the 5′-most Rap1 site and sorted by the breadth of Hmo1 binding (i.e., the number of Hmo1-containing coordinates between Rap1 and TSS). “MNase-H3” refers to dyads of H3-immunoprecipitated MNase-digested nucleosomes. A nucleotide composition plot is shown at the *right*. The *top* and *bottom* sets of panels correspond to mock heat shock and acute heat shock (5 min at 37°C), which are quantified in the bar graphs. The *bottom right* bar graphs correspond to log_2_ fold changes in Sua7/TFIIB occupancy upon heat shock at various Hmo1-width quartiles. (*B*) Frequency distribution of Hmo1 tags around Rap1 sites at RPGs having broad (*top* 30 from *A*) versus narrow (next 30) Hmo1 occupancy. The *Y*-axis is scaled to 1 for each and is oriented with the TSS to the right. (*C*) Distribution of TFIIB and RNA polymerase II (Pol II) around TSSs of subsets of RPGs having broad, narrow, and no Hmo1 (*bottom* 58 from *A*), comparing wild-type (black) and *hmo1*Δ (red) strains. The *left* panel reports on individual genes sorted as in *A*, whereas the *right* set of graphs report on the averages within each group. *n* = 30, 30, and 58 for the *top*, *middle*, and *bottom* groups. Each trace is separately scaled to 1 on the *Y*-axis. (*D*) Distribution of Fhl1 in a *hmo1*Δ strain, sorted as in *A*.

Since only about half of all RPGs contain Hmo1 (Knight et al. 2014), we took high-resolution views of factors binding individually to all 127 Rap1-bound RPGs (Fig. 2A). As quantified in Figure 2B, Hmo1 occupancy started ∼90 bp downstream from Rap1 (i.e., reached about half-maximal occupancy) and extended from ∼110 bp (“narrow”) to ∼160 bp (“broad”) further downstream, depending on the particular RPG. This width trend at individual RPGs was highly reproducible and was confirmed by other RPG-specific factors, the core transcription machinery, and histones (Fig. 2A).

Fhl1 (and FIS) displayed the same overlapping distance trend at Hmo1-enriched RPGs (Fig. 2A) but had its upstream border shifted somewhat upstream relative to Hmo1 (Fig. 2B), as reported in a prior study (Knight et al. 2014). Fhl1's downstream border fell short of Hmo1's downstream border by ∼20–30 bp except where Hmo1 had very broad binding, in which case the differential ranged up to ∼50 bp (based on visual comparisons in Supplemental Fig. S2A). These particular observations are critical for our proposed molecular mechanism of regulation. At Hmo1-deficient RPGs (Fig. 2A, bottom half of the top row of panels), Fhl1 (and FIS) covered a fixed range of ∼80 bp, instead of ∼110–160 bp. Thus, we observed two novel width modes of Fhl1 cross-linking: one fixed and one variable, with the latter being offset from Hmo1. The gene-specific trend of Hmo1 width and the downstream Fhl1/Hmo1 offset were not evident in prior ChIP-seq studies of Fhl1 and Hmo1.

Remarkably, Hmo1 interval lengths were tied to correspondingly greater distances between Rap1, the PIC (Fig. 2A, Sua7/TFIIB and Rpb3 panels; Supplemental Fig. S2A), and the +1 nucleosome (Fig. 2A, H4 and MNase-H3 panels). Except for an ∼70-bp A/T-rich gap between Rap1 and Fhl1, this suggests that nearly the entire variably lengthed RPG promoters between Rap1 and the PIC are occupied by FIS (and Hmo1, where present). In accord with a prior study (Kasahara et al. 2011), such proteins may restrict where the PIC and nucleosomes assemble.

To test the hypothesis that Hmo1 restricts the location of PIC assembly, we used ChIP-exo to map the positions of Sua7/TFIIB and RNA polymerase II (Pol II) in a *hmo1*Δ strain. Upon loss of Hmo1, Hmo1-enriched genes displayed upstream ectopic binding of TFIIB and Pol II (∼23% of the total amount recruited), spreading by as much as 200 bp upstream of the TSS (Fig. 2C, broad left shoulder in the top parts/panel). Normal placement of the remaining ∼77% of the PIC may be due to recruitment by core promoter elements and/or the +1 nucleosome. The upstream spreading was commensurate with the interval breadth of normal Hmo1 occupancy, suggesting a direct relationship and confirming single-gene experiments examining TSS and PIC shifts (Kasahara et al. 2008, 2011). In addition, a short ∼10- to 20-bp upstream shift of TFIIB and Pol II was observed at the primary PIC location. While this upstream shift appears small, in the context of the PIC structure, this may reflect a substantial post-recruitment conformational change elicited by Hmo1. As a speculative example, Hmo1 might directly or indirectly help Pol II scan downstream ∼20 bp for a TSS (Faitar et al. 2001).

Both the upstream ectopic binding and the short upstream shift of TFIIB and Pol II were not observed at RPGs that normally lacked Hmo1 (Fig. 2C, bottom panels). TFIIB and Pol II were also partially depleted at Hmo1-enriched RPG core promoters in the *hmo1*Δ strain relative to Hmo1-independent RPGs (and all other genes), with much of this being accounted for by ectopic upstream binding (Supplemental Fig. S2B). This result is consistent with Hmo1 promoting TFIID binding (Kasahara et al. 2008). PIC occupancy at RPGs that lacked Hmo1 was unaffected by Hmo1 loss, indicating that they are indeed Hmo1-independent. Taken together, these results demonstrate that the breadth of Hmo1 binding helps restrict PIC placement to RPG core promoters downstream from Rap1/FIS/Hmo1, as observed in single-gene tests (Kasahara et al. 2008, 2011).

If Hmo1 in essence promotes PIC placement, then this function might be regulated. To test this idea as well as examine the general mobilization of RPG regulators, we measured the occupancy of RPG-specific factors and the PIC under conditions of acute heat shock (5 min at 37°C), which transiently down-regulates RPG expression (Gasch et al. 2000). This represents a physiologically based and therefore regulated means of dissociating factors and assessing the consequences. The 5-min treatment represents the temporal peak of changes in factor occupancy and is likely to be too short of a time frame to incur indirect effects caused by expression changes at other genes. Upon heat shock, essentially all factors except Rap1 and Fhl1 largely dissociated (Fig. 2A, bar graphs at top and heat maps at bottom), which is consistent with single-gene studies (Cherel and Thuriaux 1995; Warner 1999; Reid et al. 2000; Mencia et al. 2002; Martin et al. 2004; Schawalder et al. 2004; Wade et al. 2004). When quantified (Fig. 2A, top bar graphs), ∼70% of Hmo1 dissociated. Both Rap1 and Fhl1 retained their exact positional organization despite the loss of most of Ifh1, Sfp1, and Hmo1. Thus, maintenance of broad patterning of Fhl1 binding over the short heat-shock period was not tied to a continued abundance of Ifh1, Sfp1, or Hmo1. As explored below, we think this is a critical observation of how RPGs are regulated.

To test directly the role of Hmo1 in Fhl1 placement, we examined the distribution of Fhl1 in a *hmo1*Δ strain. Surprisingly, we found that its normally broad distribution at Hmo1-enriched genes became constricted to the same narrow ∼80-bp interval observed at Hmo1-independent genes (Fig. 2D). This interaction was highly specific in that it was not observed at non-RPGs (data not shown) and was highly focused at a specific distance downstream from Rap1. Thus, the core binding of Fhl1 at all Rap1-regulated RPGs was Hmo1-independent. However, Hmo1 is important for the establishment but not short-term maintenance (i.e., during 5 min of heat shock) of the broad downstream extension. Since the loss of ∼70% of Hmo1 cross-linking during the acute heat shock was not accompanied by a corresponding loss of the extended Fhl1 cross-linking (i.e., no significant changes occurred) (Fig. 2A, top bar graphs), we infer that the extended Fhl1 cross-linking was not occurring through Hmo1.

### Hmo1 toggles the +1 nucleosome from a repressive to an active position

**Figure 3.**
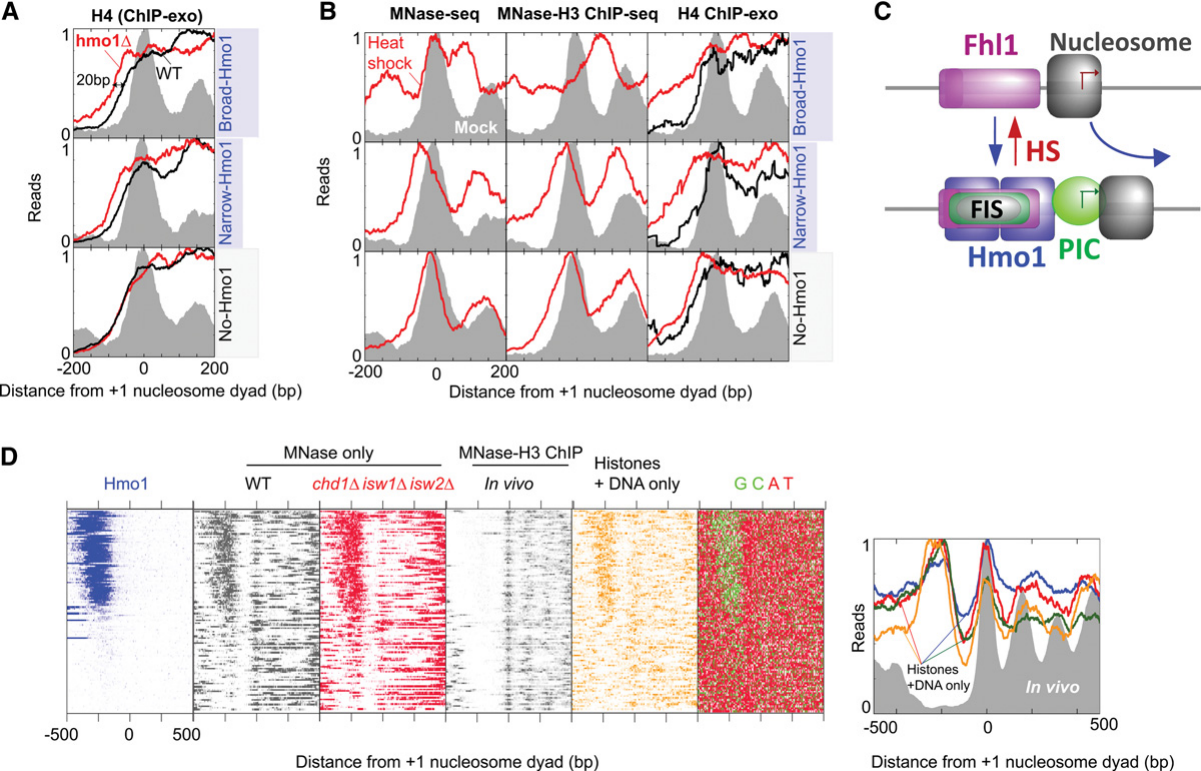
Hmo1-regulated nucleosomal organization at RPGs. (*A*) Averaged distribution of histone H4 ChIP-exo tags around the +1 nucleosome dyad of RPGs in a wild-type (black) versus a *hmo1*Δ (red) strain. The gray fill represents nucleosome dyad distributions as measured by MNase (Shivaswamy et al. 2008). The three panels represent broad, narrow, and no Hmo1 binding. (*B*) Averaged distribution of MNase-resistant DNA fragments (no ChIP vs. H3 ChIP) and H4 ChIP-exo tags around the +1 nucleosome dyad of RPGs. Gray fill and red traces represent mock and 5-min 37°C heat shock, respectively. Data in the *left* panel are from Shivaswamy et al. (2008). The *right* panel shows H4 ChIP-exo from mock (black) and heat-shocked (red) cells, with MNase-H3 ChIP nucleosomes as gray fill. (*C*) Schematic of upstream nucleosome shift upon heat shock. (*D*) Distribution of MNase-resistant fragments on individual RPGs relative to the +1 nucleosome and sorted as in Figure 2A (breadth of Hmo1 binding). The first panel is Hmo1 ChIP-exo. MNase was used in the second through fifth panels without ChIP (second and third panels) or with histone H3 ChIP (fourth and fifth panels). A+T and G+C frequencies are shown in the sixth panel. “Histones + DNA only” composite plots reflect data consistency from a variety of sources (Kaplan et al. 2009; Zhang et al. 2009, 2011b) in orange, red, blue/green, respectively.

The intimate association of the +1 nucleosome with the PIC (Rhee and Pugh 2012) led us to consider whether FIS/Hmo1 might be involved in setting the position of the +1 nucleosome. Indeed, in a *hmo1*Δ strain, the +1 nucleosome as defined by ChIP-exo of histone H4 shifted upstream by ∼20 bp on average for all Hmo1-enriched RPGs, whereas no shift was observed at Hmo1-independent genes (Fig. 3A). The magnitude of this short shift was the same as seen for the PIC in a *hmo1*Δ strain (Fig. 2C), indicating that even short movements of the PIC and +1 nucleosome are linked. An ∼20-bp shift effectively buries the canonical TSS within the nucleosome, moving it from ∼15 bp from the nucleosome edge to ∼35 bp into the nucleosome. This likely renders the TSS inaccessible to Pol II. We also examined the broad upstream region where the PIC ectopically assembled in the *hmo1*Δ strain (Fig. 2C). We failed to observe nucleosome encroachment into this region and thus surmise that the presence of the PIC prevented upstream nucleosome assembly.

We examined nucleosome repositioning when both Hmo1 and the PIC largely dissociate using a 5-min heat shock. Nucleosomes were then examined by three assays (MNase-seq, MNase-ChIP-seq, and H4 ChIP-exo) (Figs. 2A, 3B). In striking contrast to the *hmo1*Δ strain, Hmo1/PIC dissociation was accompanied by a large upstream shift of +1 nucleosome selectively at the normally Hmo1-enriched genes. Importantly, its upstream limits abutted the downstream limits of the broad distribution of Fhl1 rather than the Fhl1 core (Supplemental Fig. S2C). Thus, nucleosomes filled the region downstream from the broad Fhl1 distribution that was largely vacated by Hmo1 and the PIC.

Greater upstream nucleosome shifts were observed upon heat shock where Hmo1 normally extended more broadly beyond Fhl1 (Fig. 3B). This made the TSS less accessible and thus more repressible (Sua7/TFIIB bar graph in Fig. 2A, bottom right). In comparison, RPGs with no Hmo1 displayed nucleosomal shifts of ∼20 bp in the upstream direction (Fig. 3B, bottom panels). Their shift encompassed the region upstream of the +1 nucleosome that was vacated only by the PIC. Thus, Fhl1 is in a position to form a boundary against which the +1 nucleosome is positioned into a repressive position over the TSS upon heat shock. The position of this primary Fhl1 boundary relative to the TSS may be functionally important in that greater distances between the two were linked to greater repression (loss of Sua7/TFIIB upon heat shock) (Fig. 2A, bottom right bar graph), presumably due to increased burial of the TSS into the +1 nucleosome. In contrast, where and when present, Hmo1 and the PIC constitute a second/third barrier downstream from the first, against which the +1 nucleosome is positioned into an activating position (Fig. 3C).

### Nucleosomes are actively and constitutively excluded from RPG nucleosome-free regions (NFRs)

Many RPG NFRs are broad enough to accommodate up to two nucleosomes. Recent studies suggest that the Rap1/FIS/Hmo1 complex sits on top of nucleosomes and that nucleosomal presence in the upstream promoter region regulates RPG expression (Zeevi et al. 2011; Knight et al. 2014). In those studies, the presence of nucleosomes was generally inferred from DNA sequence features and from MNase resistance that did not involve histone ChIP. Remarkably, we found that such MNase resistance of native chromatin tracked with FIS/Hmo1 binding (Fig. 3D, first through third panels). In contrast, MNase resistance in conjunction with H3 ChIP as well as H2A, H2B, H3, and H4 ChIP-exo showed that the NFRs of RPGs were largely free of nucleosomes and histones (Figs. 2A [first and second panels], 3D [fourth panel “in vivo”]; Supplemental Fig. S3A). Moreover, heat-shock repression of RPGs was not accompanied by a general increase in nucleosome or histone occupancy in the NFRs (Fig. 2A) except for the repositioning described in Figure 3B. Such constitutive NFRs also exist during repression by nitrogen starvation (Zhang et al. 2011a).

In contrast and in accord with prior studies (Zeevi et al. 2011; Knight et al. 2014), in vitro assembled nucleosomes displayed an intrinsic preference for forming between Rap1 sites and the RPG core promoter region (Fig. 3D, fifth panel and composite plot). However, their occupancy mirrored the distribution of FIS/Hmo1, which includes an underlying nucleosome-favoring G+C enrichment (Fig. 3D, sixth panel). Thus, the wide NFRs of RPG promoters have an intrinsic preference for assembling nucleosomes but, in contrast to conclusions drawn from prior studies, are precluded from doing so in vivo by constitutively bound and MNase-resistant Fhl1 and FIS/Hmo1 complexes. As such, the large NFRs of RPGs are constitutively nucleosome-free. Such conclusions are limited to RPGs. We emphasize that, on a genomic scale, MNase-resistant fragments of nucleosomal size (and not subject to ChIP) are most likely nucleosomes, as is widely assumed. However, we found that not all MNase-resistant nucleosomal-sized fragments are nucleosomal, and thus MNase resistance without ChIP should be interpreted with caution.

Inasmuch as chromatin remodelers organize nucleosomes, we examined nucleosome organization in nine strains deleted of individual remodeler subunits (Supplemental Fig. S3B). Due to their partial functional redundancy (Gkikopoulos et al. 2011; Yen et al. 2012), effects were expected to be small. Nevertheless, loss of individual remodelers had two predominant effects. At Hmo1-enriched RPGs, loss of either Chd1, ISW2 (*isw2*Δ or *itc1*Δ), or SWI/SNF (*snf2*Δ) resulted in ∼10- to 20-bp upstream shifts of +1 nucleosomes. Thus, if Hmo1 serves as a barrier, these remodelers might normally contribute to downstream +1 positioning. In contrast, at Hmo1-independent RPGs, loss of these remodelers resulted in altered nucleosome occupancy levels (increases in the NFR and/or decreases at +1). These results suggest that multiple remodelers also contribute to normal nucleosome organization at RPGs. Analysis of the complex interplay of remodelers was beyond the scope of this study.

### DNA sequences demarcate RPG factor organization

The notion that RPG promoters tend to spatially organize RPG-specific factors in two related ways led us to follow up on prior work (Knight et al. 2014) examining their linkage to patterns of DNA sequence elements. We examined the distribution of known RPG sequence motifs for Rap1, poly(dA:dT), Fhl1, and IFHL (see Supplemental Table S1).

**Figure 4.**
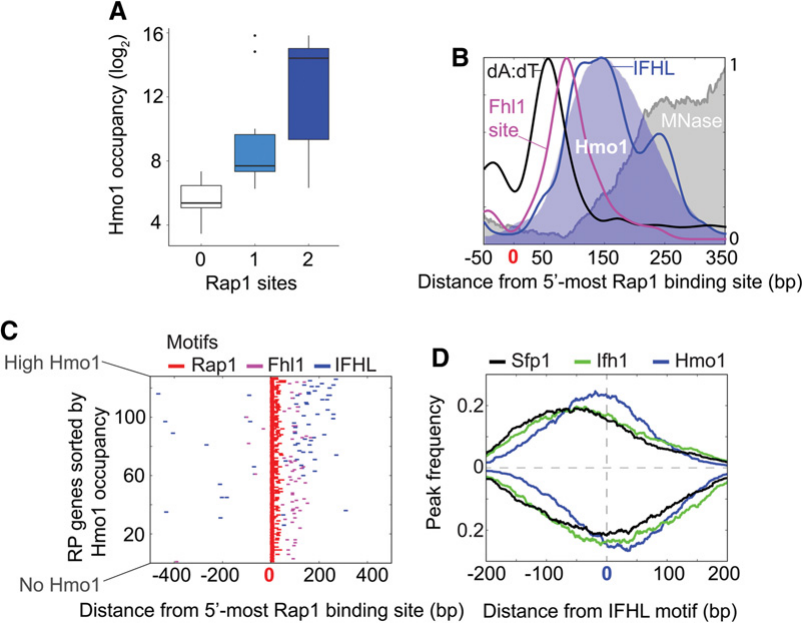
Organization of *cis*-regulatory elements at RPG promoters. (*A*) Box plot of log_2_ Hmo1 occupancy at RPGs with zero to two Rap1 sites. (*B*) Smoothed frequency distribution of >5-mer poly(dA:dT) tracts (black), Fhl1 motifs (magenta), and IFHL motifs (blue) around the most upstream Rap1 site. Nucleosomes (gray fill) and shifted 5′ end tags for Hmo1 (light-blue fill) are shown. Each trace is separately *Y*-axis-scaled to 1. (*C*) Distribution of Rap1 motifs (red; <40 bp from the primary motif), Fhl1 motifs (magenta), and IFHL motifs (blue) for each RPG around the most upstream Rap1 site, oriented with TSS to the *right* and sorted by Hmo1 occupancy. (*D*) Distribution of Sfp1 (black), Ifh1 (green), and Hmo1 (blue) ChIP-exo peak calls (GeneTrack, s5, and d20) around IFHL motifs, oriented so that RPG TSSs are to the *right*. Tag 5′ end distributions located on the antisense strand are shown inverted.

Hmo1-enriched RPGs tended to have two Rap1 sites, whereas Hmo1-independent RPGs tended toward zero or one site (Fig. 4A; Supplemental Table S2). This is consistent with Rap1 recruiting both Hmo1 (Wade et al. 2004; Hall et al. 2006; Zhao et al. 2006) and TFIID (Mencia et al. 2002; Garbett et al. 2007; Papai et al. 2010). Poly(dA:dT) tracts were enriched between Rap1 and FIS/Hmo1 (Fig. 4B), which is where nucleosomes/histones were the most depleted, as expected. Fhl1 motifs (Badis et al. 2008; Zhu et al. 2009) were positionally enriched at the Fhl1 core (i.e., defined in the *hmo1*Δ strain in Figs. 2D, 4B,C). While their positioning is consistent with a prior report (Knight et al. 2014), we found Fhl1 motifs enriched among all RPGs as opposed to just those lacking Hmo1. We attributed the difference to higher overall G+C-content of Hmo1-enriched NFRs, which makes the G+C-rich Fhl1 motif (YKYGCGTC) appear less significant compared with local sequence background and thus less detectable.

IFHL sites have been variously linked to Fhl1, Ifh1, and Hmo1. However, consistent with certain reports (Tanay et al. 2005; Hall et al. 2006; Knight et al. 2014), a strong correlation between high Hmo1 levels and the presence and positioning of IFHL sites (GGCNG) was observed (Fig. 4B,C). IFHL sites were more positionally linked to Hmo1 than to FIS (Fig. 4B,D). IFHL sites were also embedded in G+C-rich DNA sequences that mirrored the breadth of FIS/Hmo1 binding (Fig. 2A). Placing the broad G+C-rich regions through a DNA structure prediction algorithm (Zhou et al. 2013) revealed that, unlike their surrounding regions, the underlying DNA bases had a reduced propeller twist (Supplemental Fig. S4). Reduced propeller twist is associated with minor groove widening (Fratini et al. 1982), although this was only modestly indicated by DNA structure prediction (Supplemental Fig. S4). Nevertheless, a widened minor groove likely favors Hmo1 binding, since Hmo1 is a minor groove-binding protein, and thus G+C enrichment may define the breadth of Hmo1 binding, which is consistent with another report (Kasahara et al. 2011).

### Rap1 engages specific TFIID TAFs at RPGs

**Figure 5.**
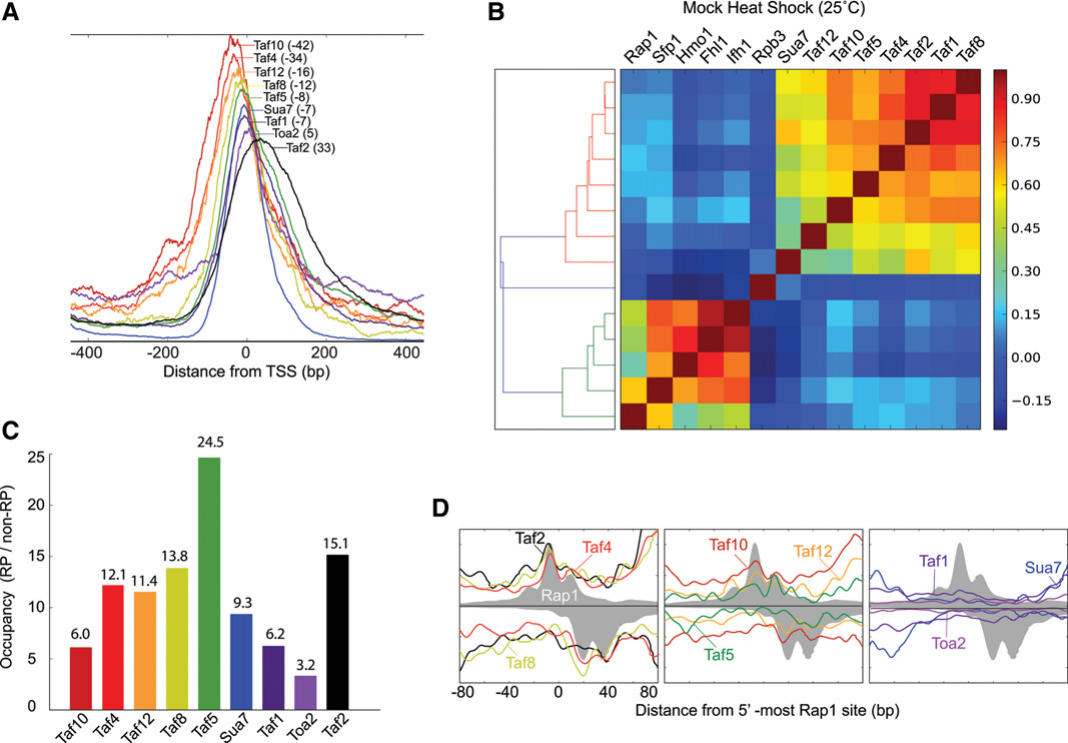
Spatial organization of TAFs in relation to TSS and Rap1. (*A*) Averaged frequency distribution of shifted tags for TAFs and general factors in relation to RPG TSSs. Peak distances from the TSS are shown in parentheses. Each trace is separately and linearly scaled on the *Y*-axis. (*B*) Heat map representing Pearson correlation values for the occupancy of proteins at RPGs. (*C*) Bar graph comparing the occupancy of Sua7, TAFs, and Toa2 at RPGs relative to non-RPGs. (*D*) Frequency distribution of unshifted tag 5′ ends for TAFs, Toa2, and Sua7 compared with the equivalent for Rap1 (filled gray plot) and oriented with the TSS to the *right*. Tags on the antisense strand are inverted.

Rap1 has been proposed to interface with TFIID via looping of the intervening DNA and in particular through Taf4, Taf5, and Taf12 TAF subunits of TFIID (Garbett et al. 2007; Layer et al. 2010). There may also be additional interactions through TFIIA/Toa2, Taf1, and Taf2 (Kasahara et al. 2008; Ohtsuki et al. 2010; Papai et al. 2010; Layer and Weil 2013). However, such interactions have been largely grounded in biochemistry or genetics rather than examination of all RPGs in their native configuration. We therefore examined the distribution of relevant TAFs and TFIIA around RPG TSSs using ChIP-exo. As expected of their core function, TAFs and TFIIA were highly enriched around the TSS (Fig. 5A). Peak-averaged occupancies ranged from 42 bp upstream (Taf10) to 33 bp downstream (Taf2), which suggest that the TFIID/A complex is spread across at least 75 bp of DNA at RPGs. This overlaps somewhat with Hmo1, which promotes TFIID binding (Kasahara et al. 2008).

Occupancy levels of TAFs and other PIC components were highly correlated with each other, as expected for an ensemble of corecruited proteins (Fig. 5B; Supplemental Table S3). Similarly, Rap1, FIS, and Hmo1 were all highly correlated with each other. However, across these two groups, the proteins were largely uncorrelated. This is consistent with FIS and Hmo1 occupancy levels being particularly related to their breadth of binding along DNA and not PIC occupancy (Fig. 2A). Pol II occupancy was largely uncorrelated with either group of proteins, which we interpret as Pol II departing into an elongating polymerase as soon as it is recruited to the promoter. Consistent with the high transcription frequency of RPGs and their known TAF dependency, RPG core promoters had high TAF occupancy compared with most other genes, with Taf5 being the most enriched (Fig. 5C). This may be due to the presence of Taf5 in both TFIID and SAGA as well has having a higher stoichiometry than some other TAFs. Note that RPGs are negatively regulated by SAGA's Gcn5 subunit via Ifh1 acetylation (Downey et al. 2013).

As with FIS, we next examined whether TAFs/TFIID, Toa2/TFIIA, and Sua7/TFIIB might cross-link at Rap1 sites to assess whether parts of the PIC may be in close proximity to Rap1. We examined the relative magnitude of cross-linking around Rap1 sites as well as how similar the patterning of tags was to that of Rap1. We observed the following hierarchy (Fig. 5D): (Taf2, Taf4, and Taf8) > (Taf5, Taf10, and Taf12) >> (Taf1, Toa2, and Sua7), with the latter having no relevant pattern around Rap1 sites. Given that differences in intrinsic cross-linkability (number, proximity, and accessibility of cross-linkable amino acids) will affect the heirarchy, distinctions between the first two groups may not be warranted. Consistent with prior reports (Garbett et al. 2007; Layer et al. 2010), Taf2, Taf4, and Taf8 (and/or Taf5, Taf10, and Taf12, some of which are heterodimeric partners) may therefore be in more direct/cross-linkable proximity with Rap1 at RPGs than other measured TAFs or general transcription factors.

### RPG coding and regulation are evolutionarily coupled

**Figure 6.**
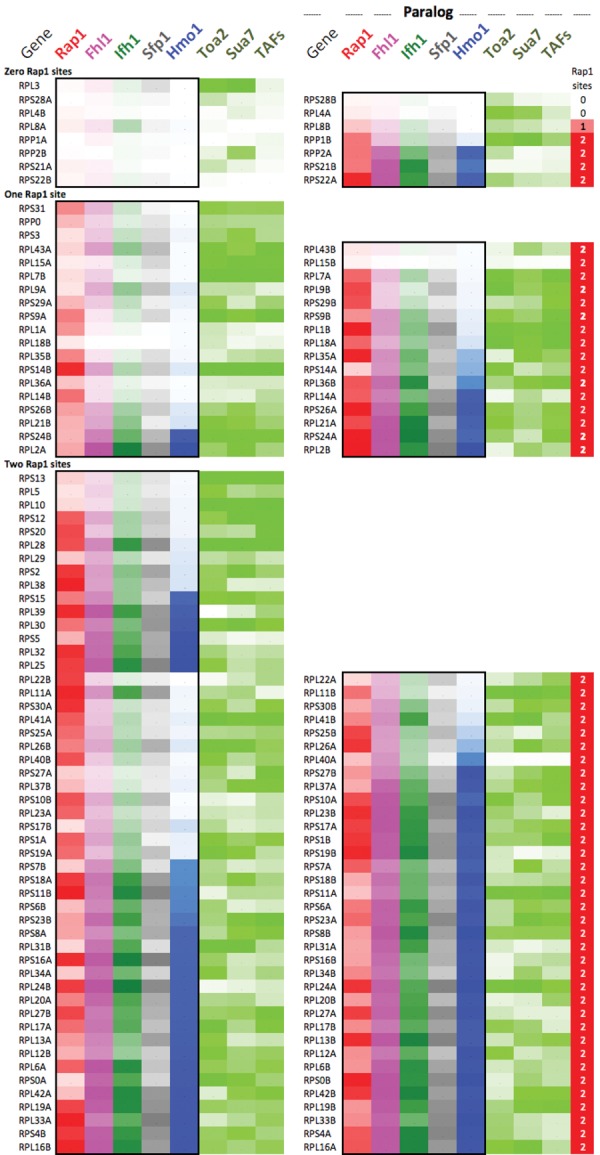
Occupancy levels at paralogous RPGs. Each row reports factor occupancy (percent rank for all but Hmo1) at paralogous RPG pairs (*left* vs. *right* set of columns). Maximum color intensity reflects a percent rank = 100.

The presence of a large number of RPGs that exist as paralogous gene pairs and as single copies provides an opportunity to examine the evolutionary divergence and conservation of RPG coregulation as related to factor occupancy. Figure 6 and Supplemental Table S2 compare factor occupancy and other properties at all 137 RPGs. RPGs were paired to their paralog and then ordered based on the number of Rap1 sites, then by the presence/absence of a paralogous gene, and finally by Hmo1 occupancy levels. RPGs lacking Rap1 sites had four evident properties: (1) They had the lowest occupancy ranks for Rap1, FIS, Hmo1, and PIC components (Supplemental Table S2, cells A2:M5). (2) They were more diverged from their paralogs in terms of both regulation and coding sequence (Supplemental Table S2, cells AJ14:AP21 and Q3:Q5). (3) They tended to lack introns (Supplemental Table S2, cells P3:P5). (4) Most importantly, they were, on average, the most represented among the group of RPGs whose kingdom-wide homologs possessed extraribosomal functions (Supplemental Table S2, cells R3:R5) (Lindstrom 2009). We infer that as certain RPGs evolved additional or different activities, their regulation also evolved to eliminate RPG-specific regulatory mechanisms.

We sought further evidence for the idea that coding and regulatory divergence are coupled by considering whether divergence of codons between paralogs was associated with increased variation in their RPG-specific factors. For consistency, we considered only RPGs where both paralogs contained two Rap1 sites. Furthermore, in this coarse-grain analysis, even paralogs having a single conservative amino acid difference were marked as nonidentical. Indeed, nonidentical paralogs had a greater differential in FIS/Hmo1 occupancy breadth than identical paralogs (Supplemental Table S2, cells AR56:AY64). This trend may represent a less-diverged state compared with RPGs with zero and one Rap1 site. Promoter regulation and coding sequence therefore appeared to be not only coevolving but also diverging from the other paralog.

Another striking observation is that, between Rap1-regulated paralogs, FIS/Hmo1 occupancies were correlated (Supplemental Table S2, cells AJ24:AQ41), as might be expected from the ancient whole-genome duplication event. In contrast, Rap1 levels and their spatial separations (in base pairs) were not correlated between paralogs, which may reflect their evolutionarily late (i.e., after whole-genome duplication and thus independent) incorporation into RP regulation (Mallick and Whiteway 2013). We speculate that specific architectures of the FIS/Hmo1 nucleosome gating mechanism (defined by the breadth of FIS/Hmo1 present) may be tied at the level of paralogous gene pairs rather than each RPG having a random version of the FIS/Hmo1 architecture.

The RP output at each paralog is expected to be half of single-copy RPGs due to equal stoichiometries of RPs in ribosomes. Consistent with this and a study by Zeevi et al. (2011), each paralog had lower PIC occupancy and correspondingly greater usage of FIS/Hmo1 than at single-copy RPGs (Supplemental Table S2, cells A7:O9). Thus, in addition to stress/nutrient-mediated control, FIS/Hmo1-directed nucleosome gating may also provide a braking mechanism that compensates for differences in gene dosage. This differs from the study by Zeevi et al. (2011), in which RPG dosage compensation was envisioned to involve upstream competitive interactions between nucleosomes and FIS as delimited by nucleosome-favoring promoter sequences.

## Discussion

**Figure 7.**
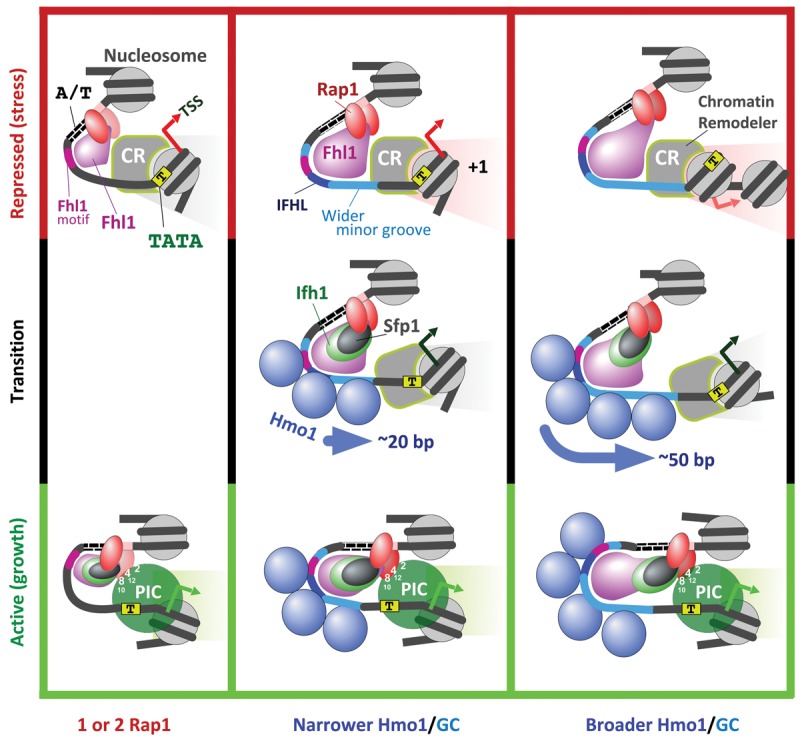
Model of RPG promoter regulation. The three columns represent three variations of the core coregulation mechanism at RPGs based on no, narrow, and broad binding of Hmo1. The *top* and *bottom* rows depict models of Rap1-mediated repression and activation, respectively. The *middle* row depicts the transition state. Relevant TAF subunit numbers are shown. The PIC reflects the general transcription machinery and a transient presence of Pol II, which rapidly moves into the elongation phase. Whether assembly or actions of the PIC also affect +1 positioning is unclear.

There are several intertwined challenges in understanding how genes are controlled: (1) identification of their regulatory factors, (2) determining how these regulatory factors are structurally organized in their native context and their mechanism of action, (3) understanding how factors coordinate their regulation with other genes, and (4) discerning whether and how evolutionary pressure is applied to gene regulation so as to be matched with evolving coding potential. The 137 RPGs of yeast are suited for this endeavor, as their regulatory factors are well known. In addition to the basic layout described previously (Knight et al. 2014), we found that the precise positional organization of RPG-specific factors, chromatin, and the transcription machinery define a binary switch for RPG transcriptional regulation. This binary switch can be adjusted by varying the downstream extension of Hmo1 binding relative to Fhl1, possibly by altering the minor groove width to which Hmo1 binds. Hmo1 toggles the switch, causing a repositioning of the +1 nucleosome from a repressive to an activating position (Fig. 7). Since the width of the switch varies from gene to gene and the unit of Hmo1 binding may be two molecules that bind ∼26 bp of DNA (Kamau et al. 2004), it is not likely to be a single monolithic structure but instead built in incremental units.

We found that nucleosomes or even histones do not assemble (i.e., <5%) over the broad RPG NFRs in vivo. Despite promoting nucleosome formation in vitro, RPG NFRs are kept broadly nucleosome-free in vivo by the constitutive presence of Rap1 (as shown originally by Yu and Morse 1999), poly(dA:dT) tracts, Fhl1 (and Hmo1, where present), and chromatin remodelers. The contiguous distribution of FIS/Hmo1 along the NFR produces the erroneous appearance of being nucleosomal, when MNase resistance rather than histone occupancy is the primary criteria for being nucleosomal.

### A molecular switch for RPG regulation

In line with early studies (Yu and Morse 1999), we propose that the constitutive binding of Rap1 to its DNA recognition sequence constitutively recruits Fhl1 to its cognate DNA sites, keeping the region constitutively nucleosome-free (Fig. 7). Under activating conditions (nutrients), Sfp1 and Ifh1 coalesce onto Fhl1, where they form the FIS complex. At half of the RPGs, the FIS complex recruits multiple Hmo1 molecules contiguously along the promoter toward the TSS, as delimited by DNA shape, which includes reduced propeller twist and minor groove widening (∼110–160 bp). Hmo1 establishes a barrier against which a chromatin remodeler positions the +1 nucleosome into an activating state adjacent to the core promoter in which it is engulfed by a FIS-enabled Rap1-recruited PIC. This involves Rap1 binding to a second Rap1 site and interacting through selected TAF subunits of TFIID (Layer et al. 2010; Papai et al. 2010). Within the activated complex, Pol II scans ∼20 bp downstream in search of a TSS just inside the +1 nucleosome. Nucleosome sliding as a potential regulatory mechanism has precedent in earlier studies (Martinez-Campa et al. 2004; Whitehouse and Tsukiyama 2006).

As well established by others using factor deletion/depletion experiments, under repressive conditions (stress or starvation), Ifh1 is sequestered away from RPGs via SAGA/Gcn5-mediated acetylation. Sfp1 is shuttled to the cytoplasm. Crf1 then binds and inactivates Fhl1. This leads to Hmo1 and PIC dissociation. Importantly, broad promoter binding of Fhl1 is retained and establishes a new barrier 20–50 bp further upstream of the TSS, against which the +1 nucleosome slides into a repressive position. At Hmo1-independent genes, there appears to be only the activating position.

### Coregulation of paralogous RPGs

Coregulation of 127 of the 137 RPGs produces remarkable unity in the spatial organization of their regulators. Those 10 RPGs that have completely eliminated the standard Rap1/FIS/Hmo1 regulation might not actually code for components of the ribosome or, if they do, may have taken on additional functions that warrant distinct regulation. In most cases, their paralog conforms to standard RPG regulation and thus may be the relevant ribosomal component.

Those RPGs that bind one Rap1 and lack a Hmo1-directed mechanism of +1 nucleosome control might also be evolving away from the paralog that uses the standard mechanism. Even where both paralogs use the standard pathway, coding and regulatory divergence are linked. As such, regulatory divergence is manifested in the breadth of FIS and Hmo1 binding at promoters. However, despite some level of divergence between paralogs, paralogous pairs tend to use the same detailed architecture of FIS/Hmo1 control.

The common thread here is that there may be two competing evolutionary forces that shape coregulation. One force imposes a single basic regulatory mechanism (Rap1/FIS/Hmo1), which varies primarily in detail among the coregulated genes (e.g., breadth of FIS and Hmo1 binding) and attempts to keep even the details fixed between paralogs. A second force attempts to diverge one member of a paralogous pair in which changes in coding and regulation coevolve. We envision “breakout” scenarios in which one paralog has acquired complete responsibility for making a ribosome subunit with the other either becoming extinct or evolving to have altered function. In the latter, pressure is exerted on the individual gene rather than on the entirety of a paralogous pair.

The work here provides a general analysis framework by which molecular mechanisms governing sets of other coregulated genes may be inferred. The framework requires having high-resolution genomic data that speak to the structural organization of nucleosomes, gene-specific factors, and general transcription factors and how they change in response to system perturbations (environmental and genetic).

## Materials and methods

### Sample preparation

*Saccharomyces cerevisiae* S288C strains were obtained from the Yeast TAP-Fusion Library (Open Biosystems). While Fhl1-TAP cross-linked to Rap1 sites, it produced a weak signal and was reported to be positionally distinct in a recent study (Knight et al. 2014). We therefore obtained the Fhl1-Myc (and Ifh1-Myc) strains from that study. Ifh1-Myc and Ifh1-TAP gave essentially identical results. Each strain was grown to OD_600_ 0.8 at 25°C in 500 mL of YPD (yeast peptone dextrose). Cells were cross-linked with 1% formaldehyde for 15 min followed by quenching with 0.125 M glycine. Heat-shocked samples were abruptly shifted for 5 min to 37°C with hot medium and then shifted back to 25°C upon cross-linking. AB9132 antibody from Abcam was used against Myc-tagged strains, while IgG Dynabeads were used against TAP-tagged strains. All experiments were performed with at least two biologically independent replicates, with each replicate allowing for the same conclusions to be drawn.

### ChIP-exo

ChIP-exo experiments were carried out essentially as described (Rhee and Pugh 2011). This included an immunoprecipitation step with magnetic beads followed by DNA polishing, A-tailing, Illumina adaptor ligation (ExA2), and λ and recJ exonuclease digestion on the beads. After elution, a primer was annealed to EXA2, extended with φ29 DNA polymerase, and then A-tailed. A second Illumina adaptor was then ligated, and the products were PCR-amplified and gel-purified. Sequencing was performed using Illumina HiSeq 2000 and NextSeq500. Uniquely aligned sequence tags were mapped to the yeast genome (sacCer3) using BWA (version 0.5.9-r16) (Li and Durbin 2009). Tags were shifted in the 3′ direction by 6 bp, and strand information was removed to better reflect the point of cross-linking.

### Peak and motif calling

MACS (Zhang et al. 2008) was used to call Rap1 peaks. Rap1 peaks in RPG promoters were subjected to MEME to obtain the consensus motif. The Rap1 consensus motif (Supplemental Table S1) was then subjected to FIMO (Find Individual Motif Occurrences) analysis (Grant et al. 2011) with default parameters and a *P*-value threshold of 0.001. All Rap1 motifs within ±40 bp of a Rap1 peak were retained. We then assigned each Rap1-binding site to the closest TSS (Xu et al. 2009) and its associated strand, if located <500 bp away.

### Occupancy

To compare mock and heat-shock data sets, data were normalized such that the total tag counts inside background regions (defined as any region outside of the 200-bp interval around a peak pair) were the same. Peaks were determined by GeneTrack (parameters: σ = 5, exclusion zone = 20) (Albert et al. 2008) and were paired if they were on opposite strands and within 100 bp in the 3′ direction. Peak pairs with more than two tag counts were considered for further analysis. Peak pairs for Ifh1, Sfp1, and Hmo1 within 400 bp upstream of RPG TSSs were subjected to MEME. Due to the high degree of overlap between different factors, the top consensus motifs discovered in the MEME analysis were Rap1, IFHL, and Poly(dA:dT) (Supplemental Table S1). The Fhl1 motif was obtained by subjecting Fhl1 peaks in *hmo1*Δ strains to MEME. FIMO analysis with default parameters and *P*-value thresholds of 0.0001 (for IFHL) and 0.001 (for Fhl1 and Poly(dA:dT) was used to obtain the motifs.

For occupancy measurements, tag counts were summed over specified intervals relative to RPG TSSs as follows: Rap1, FIS, and Hmo1 from 0 to −400 bp (upstream); Sua7/TFIIB, TFIIA/Toa2, and TAFs from −200 to +200 bp; Rpb3/Pol II from 0 to +400 bp; and Sua7 and Rpb3 in the *hmo1*Δ strain from −200 to −70 for ectopic upstream binding and −200 to +60 for total binding (for the top 30 RPGs for breadth of Hmo1 binding). Occupancies were normalized to the RPG median and log_2_-transformed, and their percent ranks were computed. Reference +1 nucleosome dyads were from Zhang et al. (2011b); TSS locations were from Xu et al. (2009).

### Accession numbers

Sequencing data are available at NCBI Sequence Read Archive under accession number SRP041518.

### Competing interest statement

B.F.P. has a financial interest in Peconic, LLC, which uses the ChIP-exo technology implemented in this study and could potentially benefit from the outcomes of this research.

## Supplementary Material

Supplemental Material

## References

[REJAGAD268896C1] Albert I, Wachi S, Jiang C, Pugh BF. 2008 GeneTrack—a genomic data processing and visualization framework. Bioinformatics 24: 1305–1306. 18388141 10.1093/bioinformatics/btn119 PMC7058423

[REJAGAD268896C2] Badis G, Chan ET, van Bakel H, Pena-Castillo L, Tillo D, Tsui K, Carlson CD, Gossett AJ, Hasinoff MJ, Warren CL, 2008 A library of yeast transcription factor motifs reveals a widespread function for Rsc3 in targeting nucleosome exclusion at promoters. Mol Cell 32: 878–887. 19111667 10.1016/j.molcel.2008.11.020 PMC2743730

[REJAGAD268896C3] Berger AB, Decourty L, Badis G, Nehrbass U, Jacquier A, Gadal O. 2007 Hmo1 is required for TOR-dependent regulation of ribosomal protein gene transcription. Mol Cell Biol 27: 8015–8026. 17875934 10.1128/MCB.01102-07 PMC2169146

[REJAGAD268896C4] Bosio MC, Negri R, Dieci G. 2011 Promoter architectures in the yeast ribosomal expression program. Transcription 2: 71–77. 21468232 10.4161/trns.2.2.14486 PMC3062397

[REJAGAD268896C5] Cherel I, Thuriaux P. 1995 The IFH1 gene product interacts with a fork head protein in *Saccharomyces cerevisiae*. Yeast 11: 261–270. 7785326 10.1002/yea.320110308

[REJAGAD268896C6] Downey M, Knight B, Vashisht AA, Seller CA, Wohlschlegel JA, Shore D, Toczyski DP. 2013 Gcn5 and sirtuins regulate acetylation of the ribosomal protein transcription factor Ifh1. Curr Biol 23: 1638–1648. 23973296 10.1016/j.cub.2013.06.050 PMC3982851

[REJAGAD268896C7] Faitar SL, Brodie SA, Ponticelli AS. 2001 Promoter-specific shifts in transcription initiation conferred by yeast TFIIB mutations are determined by the sequence in the immediate vicinity of the start sites. Mol Cell Biol 21: 4427–4440. 11416123 10.1128/MCB.21.14.4427-4440.2001 PMC87103

[REJAGAD268896C8] Fratini AV, Kopka ML, Drew HR, Dickerson RE. 1982 Reversible bending and helix geometry in a B-DNA dodecamer: CGCGAATTBrCGCG. J Biol Chem 257: 14686–14707. 7174662

[REJAGAD268896C9] Garbett KA, Tripathi MK, Cencki B, Layer JH, Weil PA. 2007 Yeast TFIID serves as a coactivator for Rap1p by direct protein-protein interaction. Mol Cell Biol 27: 297–311. 17074814 10.1128/MCB.01558-06 PMC1800639

[REJAGAD268896C10] Gasch AP, Spellman PT, Kao CM, Carmel-Harel O, Eisen MB, Storz G, Botstein D, Brown PO. 2000 Genomic expression programs in the response of yeast cells to environmental changes. Mol Biol Cell 11: 4241–4257. 11102521 10.1091/mbc.11.12.4241 PMC15070

[REJAGAD268896C11] Gkikopoulos T, Schofield P, Singh V, Pinskaya M, Mellor J, Smolle M, Workman JL, Barton GJ, Owen-Hughes T. 2011 A role for Snf2-related nucleosome-spacing enzymes in genome-wide nucleosome organization. Science 333: 1758–1760. 21940898 10.1126/science.1206097 PMC3428865

[REJAGAD268896C12] Grant CE, Bailey TL, Noble WS. 2011 FIMO: scanning for occurrences of a given motif. Bioinformatics 27: 1017–1018. 21330290 10.1093/bioinformatics/btr064 PMC3065696

[REJAGAD268896C13] Hall DB, Wade JT, Struhl K. 2006 An HMG protein, Hmo1, associates with promoters of many ribosomal protein genes and throughout the rRNA gene locus in *Saccharomyces cerevisiae*. Mol Cell Biol 26: 3672–3679. 16612005 10.1128/MCB.26.9.3672-3679.2006 PMC1447432

[REJAGAD268896C14] Joo YJ, Kim JH, Kang UB, Yu MH, Kim J. 2011 Gcn4p-mediated transcriptional repression of ribosomal protein genes under amino-acid starvation. EMBO J 30: 859–872. 21183953 10.1038/emboj.2010.332 PMC3049204

[REJAGAD268896C15] Jorgensen P, Rupes I, Sharom JR, Schneper L, Broach JR, Tyers M. 2004 A dynamic transcriptional network communicates growth potential to ribosome synthesis and critical cell size. Genes Dev 18: 2491–2505. 15466158 10.1101/gad.1228804 PMC529537

[REJAGAD268896C16] Kamau E, Bauerle KT, Grove A. 2004 The *Saccharomyces cerevisiae* high mobility group box protein HMO1 contains two functional DNA binding domains. J Biol Chem 279: 55234–55240. 15507436 10.1074/jbc.M409459200

[REJAGAD268896C17] Kaplan N, Moore IK, Fondufe-Mittendorf Y, Gossett AJ, Tillo D, Field Y, LeProust EM, Hughes TR, Lieb JD, Widom J, 2009 The DNA-encoded nucleosome organization of a eukaryotic genome. Nature 458: 362–366. 19092803 10.1038/nature07667 PMC2658732

[REJAGAD268896C18] Kasahara K, Ohtsuki K, Ki S, Aoyama K, Takahashi H, Kobayashi T, Shirahige K, Kokubo T. 2007 Assembly of regulatory factors on rRNA and ribosomal protein genes in *Saccharomyces cerevisiae*. Mol Cell Biol 27: 6686–6705. 17646381 10.1128/MCB.00876-07 PMC2099245

[REJAGAD268896C19] Kasahara K, Ki S, Aoyama K, Takahashi H, Kokubo T. 2008 *Saccharomyces cerevisiae* HMO1 interacts with TFIID and participates in start site selection by RNA polymerase II. Nucleic Acids Res 36: 1343–1357. 18187511 10.1093/nar/gkm1068 PMC2275077

[REJAGAD268896C20] Kasahara K, Ohyama Y, Kokubo T. 2011 Hmo1 directs pre-initiation complex assembly to an appropriate site on its target gene promoters by masking a nucleosome-free region. Nucleic Acids Res 39: 4136–4150. 21288884 10.1093/nar/gkq1334 PMC3105432

[REJAGAD268896C21] Knight B, Kubik S, Ghosh B, Bruzzone MJ, Geertz M, Martin V, Denervaud N, Jacquet P, Ozkan B, Rougemont J, 2014 Two distinct promoter architectures centered on dynamic nucleosomes control ribosomal protein gene transcription. Genes Dev 28: 1695–1709. 25085421 10.1101/gad.244434.114 PMC4117944

[REJAGAD268896C22] Kurtz S, Shore D. 1991 RAP1 protein activates and silences transcription of mating-type genes in yeast. Genes Dev 5: 616–628. 2010087 10.1101/gad.5.4.616

[REJAGAD268896C23] Layer JH, Weil PA. 2013 Direct TFIIA–TFIID protein contacts drive budding yeast ribosomal protein gene transcription. J Biol Chem 288: 23273–23294. 23814059 10.1074/jbc.M113.486829 PMC3743499

[REJAGAD268896C24] Layer JH, Miller SG, Weil PA. 2010 Direct transactivator–transcription factor IID (TFIID) contacts drive yeast ribosomal protein gene transcription. J Biol Chem 285: 15489–15499. 20189987 10.1074/jbc.M110.104810 PMC2865315

[REJAGAD268896C25] Li H, Durbin R. 2009 Fast and accurate short read alignment with Burrows-Wheeler transform. Bioinformatics 25: 1754–1760. 19451168 10.1093/bioinformatics/btp324 PMC2705234

[REJAGAD268896C26] Li B, Nierras CR, Warner JR. 1999 Transcriptional elements involved in the repression of ribosomal protein synthesis. Mol Cell Biol 19: 5393–5404. 10409730 10.1128/mcb.19.8.5393 PMC84382

[REJAGAD268896C27] Lieb JD, Liu X, Botstein D, Brown PO. 2001 Promoter-specific binding of Rap1 revealed by genome-wide maps of protein–DNA association. Nat Genet 28: 327–334. 11455386 10.1038/ng569

[REJAGAD268896C28] Lindstrom MS. 2009 Emerging functions of ribosomal proteins in gene-specific transcription and translation. Biochem Biophys Res Commun 379: 167–170. 19114035 10.1016/j.bbrc.2008.12.083

[REJAGAD268896C29] Mallick J, Whiteway M. 2013 The evolutionary rewiring of the ribosomal protein transcription pathway modifies the interaction of transcription factor heteromer Ifh1–Fhl1 (interacts with forkhead 1–forkhead-like 1) with the DNA-binding specificity element. J Biol Chem 288: 17508–17519. 23625919 10.1074/jbc.M112.436683 PMC3682550

[REJAGAD268896C30] Marion RM, Regev A, Segal E, Barash Y, Koller D, Friedman N, O'Shea EK. 2004 Sfp1 is a stress- and nutrient-sensitive regulator of ribosomal protein gene expression. Proc Natl Acad Sci 101: 14315–14322. 15353587 10.1073/pnas.0405353101 PMC521938

[REJAGAD268896C31] Martin DE, Soulard A, Hall MN. 2004 TOR regulates ribosomal protein gene expression via PKA and the Forkhead transcription factor FHL1. Cell 119: 969–979. 15620355 10.1016/j.cell.2004.11.047

[REJAGAD268896C32] Martinez-Campa C, Politis P, Moreau JL, Kent N, Goodall J, Mellor J, Goding CR. 2004 Precise nucleosome positioning and the TATA box dictate requirements for the histone H4 tail and the bromodomain factor Bdf1. Mol Cell 15: 69–81. 15225549 10.1016/j.molcel.2004.05.022

[REJAGAD268896C33] Mencia M, Moqtaderi Z, Geisberg JV, Kuras L, Struhl K. 2002 Activator-specific recruitment of TFIID and regulation of ribosomal protein genes in yeast. Mol Cell 9: 823–833. 11983173 10.1016/s1097-2765(02)00490-2

[REJAGAD268896C34] Ohtsuki K, Kasahara K, Shirahige K, Kokubo T. 2010 Genome-wide localization analysis of a complete set of Tafs reveals a specific effect of the taf1 mutation on Taf2 occupancy and provides indirect evidence for different TFIID conformations at different promoters. Nucleic Acids Res 38: 1805–1820. 20026583 10.1093/nar/gkp1172 PMC2847235

[REJAGAD268896C35] Papai G, Tripathi MK, Ruhlmann C, Layer JH, Weil PA, Schultz P. 2010 TFIIA and the transactivator Rap1 cooperate to commit TFIID for transcription initiation. Nature 465: 956–960. 20559389 10.1038/nature09080 PMC2900199

[REJAGAD268896C36] Reid JL, Iyer VR, Brown PO, Struhl K. 2000 Coordinate regulation of yeast ribosomal protein genes is associated with targeted recruitment of Esa1 histone acetylase. Mol Cell 6: 1297–1307. 11163204 10.1016/s1097-2765(00)00128-3

[REJAGAD268896C37] Rhee HS, Pugh BF. 2011 Comprehensive genome-wide protein-DNA interactions detected at single-nucleotide resolution. Cell 147: 1408–1419. 22153082 10.1016/j.cell.2011.11.013 PMC3243364

[REJAGAD268896C38] Rhee HS, Pugh BF. 2012 Genome-wide structure and organization of eukaryotic pre-initiation complexes. Nature 483: 295–301. 22258509 10.1038/nature10799 PMC3306527

[REJAGAD268896C39] Rudra D, Zhao Y, Warner JR. 2005 Central role of Ifh1p–Fhl1p interaction in the synthesis of yeast ribosomal proteins. EMBO J 24: 533–542. 15692568 10.1038/sj.emboj.7600553 PMC548658

[REJAGAD268896C40] Schawalder SB, Kabani M, Howald I, Choudhury U, Werner M, Shore D. 2004 Growth-regulated recruitment of the essential yeast ribosomal protein gene activator Ifh1. Nature 432: 1058–1061. 15616569 10.1038/nature03200

[REJAGAD268896C41] Shivaswamy S, Bhinge A, Zhao Y, Jones S, Hirst M, Iyer VR. 2008 Dynamic remodeling of individual nucleosomes across a eukaryotic genome in response to transcriptional perturbation. PLoS Biol 6: e65. 18351804 10.1371/journal.pbio.0060065 PMC2267817

[REJAGAD268896C42] Shore D. 1994 RAP1: a protean regulator in yeast. Trends Genet 10: 408–412. 7809947 10.1016/0168-9525(94)90058-2

[REJAGAD268896C43] Tanay A, Regev A, Shamir R. 2005 Conservation and evolvability in regulatory networks: the evolution of ribosomal regulation in yeast. Proc Natl Acad Sci 102: 7203–7208. 15883364 10.1073/pnas.0502521102 PMC1091753

[REJAGAD268896C44] Wade JT, Hall DB, Struhl K. 2004 The transcription factor Ifh1 is a key regulator of yeast ribosomal protein genes. Nature 432: 1054–1058. 15616568 10.1038/nature03175

[REJAGAD268896C45] Wapinski I, Pfiffner J, French C, Socha A, Thompson DA, Regev A. 2010 Gene duplication and the evolution of ribosomal protein gene regulation in yeast. Proc Natl Acad Sci 107: 5505–5510. 20212107 10.1073/pnas.0911905107 PMC2851827

[REJAGAD268896C46] Warner JR. 1999 The economics of ribosome biosynthesis in yeast. Trends Biochem Sci 24: 437–440. 10542411 10.1016/s0968-0004(99)01460-7

[REJAGAD268896C47] Whitehouse I, Tsukiyama T. 2006 Antagonistic forces that position nucleosomes in vivo. Nat Struct Mol Biol 13: 633–640. 16819518 10.1038/nsmb1111

[REJAGAD268896C48] Wolfe KH, Shields DC. 1997 Molecular evidence for an ancient duplication of the entire yeast genome. Nature 387: 708–713. 9192896 10.1038/42711

[REJAGAD268896C49] Xiao L, Kamau E, Donze D, Grove A. 2011 Expression of yeast high mobility group protein HMO1 is regulated by TOR signaling. Gene 489: 55–62. 21924331 10.1016/j.gene.2011.08.017

[REJAGAD268896C50] Xu Z, Wei W, Gagneur J, Perocchi F, Clauder-Munster S, Camblong J, Guffanti E, Stutz F, Huber W, Steinmetz LM. 2009 Bidirectional promoters generate pervasive transcription in yeast. Nature 457: 1033–1037. 19169243 10.1038/nature07728 PMC2766638

[REJAGAD268896C51] Yen K, Vinayachandran V, Batta K, Koerber RT, Pugh BF. 2012 Genome-wide nucleosome specificity and directionality of chromatin remodelers. Cell 149: 1461–1473. 22726434 10.1016/j.cell.2012.04.036 PMC3397793

[REJAGAD268896C52] Yu L, Morse RH. 1999 Chromatin opening and transactivator potentiation by RAP1 in *Saccharomyces cerevisiae*. Mol Cell Biol 19: 5279–5288. 10409719 10.1128/mcb.19.8.5279 PMC84371

[REJAGAD268896C53] Zeevi D, Sharon E, Lotan-Pompan M, Lubling Y, Shipony Z, Raveh-Sadka T, Keren L, Levo M, Weinberger A, Segal E. 2011 Compensation for differences in gene copy number among yeast ribosomal proteins is encoded within their promoters. Genome Res 21: 2114–2128. 22009988 10.1101/gr.119669.110 PMC3227101

[REJAGAD268896C54] Zhang Y, Liu T, Meyer CA, Eeckhoute J, Johnson DS, Bernstein BE, Nusbaum C, Myers RM, Brown M, Li W, 2008 Model-based analysis of ChIP-seq (MACS). Genome Biol 9: R137. 18798982 10.1186/gb-2008-9-9-r137 PMC2592715

[REJAGAD268896C55] Zhang Y, Moqtaderi Z, Rattner BP, Euskirchen G, Snyder M, Kadonaga JT, Liu XS, Struhl K. 2009 Intrinsic histone–DNA interactions are not the major determinant of nucleosome positions in vivo. Nat Struct Mol Biol 16: 847–852. 19620965 10.1038/nsmb.1636 PMC2823114

[REJAGAD268896C56] Zhang L, Ma H, Pugh BF. 2011a Stable and dynamic nucleosome states during a meiotic developmental process. Genome Res 21: 875–884. 21515815 10.1101/gr.117465.110 PMC3106320

[REJAGAD268896C57] Zhang Z, Wippo CJ, Wal M, Ward E, Korber P, Pugh BF. 2011b A packing mechanism for nucleosome organization reconstituted across a eukaryotic genome. Science 332: 977–980. 21596991 10.1126/science.1200508 PMC4852979

[REJAGAD268896C58] Zhao Y, McIntosh KB, Rudra D, Schawalder S, Shore D, Warner JR. 2006 Fine-structure analysis of ribosomal protein gene transcription. Mol Cell Biol 26: 4853–4862. 16782874 10.1128/MCB.02367-05 PMC1489154

[REJAGAD268896C59] Zhou T, Yang L, Lu Y, Dror I, Dantas Machado AC, Ghane T, Di Felice R, Rohs R. 2013 DNAshape: a method for the high-throughput prediction of DNA structural features on a genomic scale. Nucleic Acids Res 41: W56–W62. 23703209 10.1093/nar/gkt437 PMC3692085

[REJAGAD268896C60] Zhu C, Byers KJ, McCord RP, Shi Z, Berger MF, Newburger DE, Saulrieta K, Smith Z, Shah MV, Radhakrishnan M, 2009 High-resolution DNA-binding specificity analysis of yeast transcription factors. Genome Res 19: 556–566. 19158363 10.1101/gr.090233.108 PMC2665775

