# Supplementary material for: Molecular mechanisms of ribosomal protein gene coregulation
Source: Genes Dev. 2015 Sep 15;29(18):1942–54. doi: 10.1101/gad.268896.115 (PMC4579351; doi:10.1101/gad.268896.115)
Supplement: Supplemental Material [file supp_29_18_1942__index.html]

Supplemental Material 

# Molecular mechanisms of ribosomal protein gene coregulation

## Supplemental Material

**Files in this Data Supplement:**

- Supp Material.docx
- Supp Table S1.xlsx
- Supp Table S2.xlsx
- Supp Table S3.xlsx
