# Supplementary material for: Molecular mechanisms of ribosomal protein gene coregulation
Source: Genes Dev. 2015 Sep 15;29(18):1942–54. doi: 10.1101/gad.268896.115 (PMC4579351; doi:10.1101/gad.268896.115)
Supplement: Supplemental Material [file supp_29.18.1942_SuppMaterial.docx]

Supplementary Information

**SUPPLEMENTAL FIGURES**

**Figure S1. Spatial Organization of Rap1-bound Sites at RPGs, related to Figure 1**

**Figure S2. Relative Occupancy of TFIIB and Pol II at RPGs in Wild Type and *hmo1Δ* strains, related to Figure 2**

**Figure S3. Distribution of Rap1 and Histones around the 5’-most Rap1 sites, related to Figure 3**

**Figure S4. Distribution of DNA Structural Features around the 5’-most Rap1 site, related to Figure 4**

**SUPPLEMENTAL TABLES**

**Table S1: Position-specific probability matrix (PSPM) for Rap1, Ifh1, Fhl1 and Poly-A motif, related to Figure 1, Figure 4, and Figure S1.**

**Table S2: Comparison of factor occupancy and other properties of 137 RPG promoters, related to Figure 6.**

**Table S3: Pairwise occupancy correlation between RPG factors and general transcription factors, related to Figure 5B.**

**SUPPLEMENTAL REFERENCES**

**Figure S1. Spatial organization of Rap1-bound sites at RPGs, related to Figure 1**

Panel 1: Nucleotide composition (colors) of 127 RPG Rap1-bound regions (rows), aligned by the most upstream Rap1 site, orientated with the TSS to the right, and sorted by distance between adjacent Rap1 sites. Panel 2: Corresponding ChIP-exo tag 5’ ends (zoomed out), located on the same (blue) or opposite (red) strand as the TSS, are plotted as an overlay. Panels 3 and 4: Same as panel 2, except data are not overlayed, and are aligned to all 234 Rap1 motifs at RPGs, and sorted by motif strength. A schematic of the ChIP-exo assay is shown.

**Figure S2. Relative Occupancy of TFIIB and Pol II at RPGs in Wild Type and *hmo1Δ* strains, related to Figure 2**

**(A)** Heat map showing shifted ChIP-exo tag 5’-ends for Hmo1 (blue), Fhl1 (magenta), and Sua7/TFIIB (green) aligned by the 5’-most Rap1 site, and sorted by Hmo1 occupancy. Overlapping regions are colored black.

**(B)** Occupancy of Sua7 and Rpb3 around TSSs of subsets of RPGs, either having or lacking Hmo1 binding (top 60 and bottom 58 of Hmo1-occupied RPGs, respectively), comparing wild type (black and gray) and *hmo1Δ* (orange and red) strains.

**(C)** Distribution of Fhl1 in wild type heat shocked cells (37˚C, 5 min, magenta in left panel), or in *hmo1Δ* cells (no heat shock, representing the core binding in darker magenta, right panel). Overlayed on these panels are individual MNase H3-ChIP-seq tags, representing nucleosome dyads measured upon acute heat shock (37˚C, 5 min, black). All locations are aligned by the 5’-most Rap1 site and sorted by Hmo1 occupancy as in panel **(A)**.

**Figure S3. Distribution of Rap1 and Histones around the 5’-most Rap1 sites, related to Figure 3**

**(A)** Top panel: Heat map showing shifted ChIP-exo tag 5’-ends for Rap1 and histones, aligned by the upstream most Rap1 site and sorted by Rap1 occupancy. Bottom panel: Averaged distribution of histone ChIP-exo tags at the top and bottom 25% of Rap1-bound RPGs (darker and lighter colored traces, respectively). The distribution of Rap1 tags is shown as a red fill. These results are similar to those shown by Lickwar and Leib (Lickwar et al. 2013).

**(B)** Top panel: Averaged distribution of nucleosomes defined by MNase-H3 ChIP for BY4742 wild type (gray fill) and remodeler subunit deletion mutants (red trace), around TSSs of subsets of RPG having Hmo1 and no Hmo1 (n = 60, 58 for top and bottom panels, respectively). Bottom panel: Heat map showing shifted tag 5’-ends for BY4742 (blue) and *isw2Δ* and *snf2Δ* (red) strains around RPG TSSs, sorted by Hmo1 occupancy. Data for both panels are from (Yen et al. 2012).

**Figure S4. Distribution of DNA Structural Features around the 5’-most Rap1 site, related to Figure 4**

DNA structural features (propeller twist, helical twist, minor groove width and roll) around the 5’-most Rap1 sites were sorted by Hmo1 occupancy with TSS oriented to the right. DNA structural features were obtained using DNA shape software (Zhou et al. 2013). Scrambling the DNA sequence within Hmo1-bound regions, which maintained overall G+C-richness, predicted less reduction in propeller twist (not shown).

**SUPPLEMENTAL REFERENCES**

Lickwar CR, Mueller F, Lieb JD. 2013. Genome-wide measurement of protein-DNA binding dynamics using competition ChIP. *Nature protocols* **8**: 1337-1353.

Yen K, Vinayachandran V, Batta K, Koerber RT, Pugh BF. 2012. Genome-wide Nucleosome Specificity and Directionality of Chromatin Remodelers. *Cell* **149**: 1461-1473.

Zhou T, Yang L, Lu Y, Dror I, Dantas Machado AC, Ghane T, Di Felice R, Rohs R. 2013. DNAshape: a method for the high-throughput prediction of DNA structural features on a genomic scale. *Nucleic Acids Res* **41**: W56-62.
